# Association of childhood externalizing, internalizing and comorbid symptoms with long-term economic outcomes

**DOI:** 10.1192/j.eurpsy.2022.1102

**Published:** 2022-09-01

**Authors:** F. Vergunst, M. Commisso, M.-C. Geoffroy, C. Temcheff, S. Scardera, M. Poirier, S. Côté, F. Vitaro, G. Turecki, R. Tremblay, M. Orri

**Affiliations:** 1 University of Montreal, Social And Preventive Medicine, Chemin de la Côte Ste-Catherine , Canada; 2 McGill University, Department Of Psychiatry, Rue Sherbrooke, Canada; 3 McGill University, Department Of Educational And Counselling Psychology, Rue Sherbrooke, Canada; 4 University of Montreal, Department Of Psychoeducation, Chemin de la Côte Ste-Catherine , Canada

**Keywords:** disruptive behaviors, developmental psychopathology, behavioral disorders

## Abstract

**Introduction:**

Externalising and internalising problems are common in school-aged children. Few studies have examined the association between comorbid externalising and internalising symptoms and adult-life economic participation.

**Objectives:**

To investigate associations of childhood externalising, internalising, and comorbid internalising-externalising symptoms with earnings and welfare receipt in adulthood.

**Methods:**

We used group-based trajectory modeling to identify profiles of children with externalising, internalising, and comorbid symptoms from age 6-12 years. We estimated associations of the identified profiles with participants’ employment earnings at age 33-37 years and welfare receipt from age 18-35 years obtained from tax return records. The child’s IQ and family socioeconomic background were adjusted for.

**Results:**

Four profiles were identified: no symptoms (45%), externalizing (29%), internalizing (11%) and comorbid symptoms (13%). Relative to the no-symptom profile, participants in the comorbid profile earned US$-18,323 less annually (95%CI=-20,925 to -15700) at age 33-37 years and were significantly more likely to receive welfare across follow-up (RR=6.30, 95%CI=5.4 to 7.2). Similarly, compared to the no-symptom profile, participants in the externalising profile earned US$-7,256 less per year (95%CI=-9,205 to -5,307), while participants in the internalising profile earned US$-9,716 less (95%CI=-12,358 to -7,074). Significant interactions by sex were observed. For participants in the comorbid profile, males were more likely to have lower earnings while females were more likely to receive welfare, relative to the no-symptom profile.

**Conclusions:**

Children exhibiting comorbid externalising and internalising symptoms are at high risk of poor economic outcomes in adulthood. Early detection, prevention and management is crucial to improve the life chances of this vulnerable population.

**Disclosure:**

No significant relationships.

